# Interobserver variability of assessing body condition scores and muscle condition scores in a population of 43 active working explosive detection dogs

**DOI:** 10.3389/fvets.2024.1431855

**Published:** 2024-10-17

**Authors:** Kimberly M. Christie, Jennifer A. Barnhard, Cynthia M. Otto, Amritha Mallikarjun, Clara Wilson, David Levine, Ashley A. Tringali, Chelsea E. Payne, Anke Langenbach, Matthew W. Brunke

**Affiliations:** ^1^Veterinary Surgical Centers, Vienna, VA, United States; ^2^Tactical Veterinary Solutions LLC, Cabin John, MD, United States; ^3^Penn Vet Working Dog Center, Clinical Sciences and Advanced Medicine, School of Veterinary Medicine, University of Pennsylvania, Philadelphia, PA, United States; ^4^Department of Physical Therapy, University of Tennessee at Chattanooga, Chattanooga, TN, United States; ^5^U.S. Army Veterinary Corps, Fort Belvoir, VA, United States; ^6^Veterinary Referral Associates, Gaithersburg, MD, United States

**Keywords:** BCS, MCS, detection dog, EDD, handler, working dog, veterinarian

## Abstract

**Objectives:**

This study aimed to evaluate the agreement between explosive detection dog (EDD) handlers and a team of veterinarians in assessing body condition score (BCS) and muscle condition score (MCS), hypothesizing significant BCS differences between handlers and veterinarians, and no significant MCS differences in healthy active duty EDDs.

**Methods:**

This prospective study analyzed variance and inter-rater intraclass correlation coefficients (ICC) of agreement within BCS and MCS assessments collected from the 43 EDDs by four blinded graders; the EDDs’ respective handler and three veterinarians with varying levels of veterinary expertise.

**Results:**

The results of the study showed that 74.4% of the EDD population was graded as ideal BCS (4 or 5 out of 9) by the handlers compared to 67.44% by the members of the veterinary team; however, the graders scored different subsets of individual EDDs as ideal. Normal MCS (3 out of 3) was assessed in 86.05% (*n* = 37) of EDDs by the handlers versus in 70.54% by the veterinary team.

**Conclusion:**

This study highlights the importance of standardized training and guidelines for BCS and MCS assessments in working dogs to improve agreement between all members of the healthcare team.

## Introduction

Explosive detection dogs (EDDs) play a critical role in detecting and signaling the presence of explosive materials to their handlers (1, 2). Operating in diverse, demanding, and often public-facing environments (e.g., war zones, sports arenas, and transportation hubs), EDDs must maintain peak levels of health and physical fitness to optimally perform their jobs (3, 4). Historically, traditional clinical assessments have relied on body weight to determine and monitor canine health and fitness (5, 6). However, recent attention has shifted to the body condition score (BCS) and, in some cases, muscle condition score (MCS), which offer valuable insights into the balance between body fat and lean muscle composition (4, 7).

Regular assessment and monitoring of BCS, MCS, and body weight (BW) plays a crucial role in detecting, managing, and preventing adverse health effects associated with an imbalance of fat and muscle (8–11). An elevated BCS, for example, has been associated with an array of health issues, including musculoskeletal conditions, endocrine and cardiovascular diseases, neoplastic processes, and a shortened working career and overall life expectancy (4, 12–15). Additionally, overweight and obese dogs, which exhibit higher internal body temperatures and tend to pant as a thermoregulatory response, experience reduced olfaction efficiency because panting prevents them from sniffing simultaneously (16–19). This could have significant consequences given the vital role these dogs serve in public safety.

Objective measurements of BCS and MCS typically involve techniques such as dual-energy X-ray absorptiometry (DEXA), computed tomography (CT), quantitative magnetic resonance (QMR), and ultrasound (8, 10, 20, 21). However, these methods often require costly specialized equipment, specific expertise, or the use of anesthesia, rendering them impractical for working dog handlers or standard veterinary practices (10, 13, 21). Consequently, there is a need for an efficient, affordable, and semi-quantitative method for assessing BCS and MCS. This method should ensure consistent agreement among handlers and veterinary professionals, facilitating effective communication of health and fitness changes to sustain optimal performance.

Scoring of body condition and muscle condition involves both palpation and visual assessment using developed scales to gauge levels of external body fat and lean muscle tissue (8, 22–24). Several validated scoring systems have been utilized to assess BCS including a 5-point scale and a 9-point scale, with the 9-point scale being most common due to its established correlation with DEXA (7, 14, 15, 24, 25). An optimal BCS for dogs on the 9-point scale is 4 to 5, with research suggesting that working dogs may benefit from having a BCS on the lower end of ideal (4, 7, 24). The MCS system, introduced by the World Small Animal Veterinary Association (WSAVA), assesses muscle loss, using a scale ranging from ‘normal musculature’ to ‘marked muscle atrophy’ (8). However, the MCS system for dogs currently lacks validation. There is a validated MCS scale for cats which uses a numerical scale ranging from 0 to 3 to indicate the degree of muscle atrophy, with 3 indicating normal musculature although it lacks precise boundaries between categories (9). Despite this limitation, the MCS system is still utilized to subjectively evaluate muscle atrophy resulting from conditions such as sarcopenia or cachexia (9, 10). Both BCS and MCS, when used in combination with a physical examination, can be valuable tools in helping to evaluate a dog’s overall physical health and working potential.

While the 9-point BCS scale aims for universal usability (26), there remains a discrepancy in accurately gauging a dog’s BCS among individuals with varying levels of veterinary expertise (27–31). Prior research studies found between 44 and 65% of pet and sporting dog owners frequently encounter difficulties accurately gauging their dog’s body condition and often underestimate it, especially in cases of overweight dogs (27–31). When evaluating the level of agreement between pet owners and veterinary professionals in determining the BCS of overweight dogs, the analysis shows only a 53% agreement, with 39% of owners rating their overweight dogs as having an ideal BCS (32). Studies evaluating the agreement between individuals assessing MCS have been reported in cats but are sparse in number and have reported agreement between individuals within the veterinary field but not between owners and veterinary professionals (9). There are currently no studies that assess the level of agreement between owners and veterinary professionals on MCS in dogs.

Despite the growing recognition of the importance of BCS and MCS in companion, working, and sporting dog health assessments, there is limited to no research on the agreement between handlers and veterinary professionals in reporting BCS and MCS in working dogs. This study aims to fill this gap by assessing the level of agreement among handlers and a team of veterinarians in grading the BCS and MCS of an active working dog population. We hypothesized that there would be significant differences in reported BCS between handlers and veterinarians, as well as between primary care veterinarians and sports medicine-focused veterinarians. Additionally, we anticipate no significant differences in reported MCS between the graders as it is unlikely to find muscle atrophy in active duty working dogs, leading to minimal variability between grader scores.

## Materials and methods

### Study design and participants

This prospective study analyzed the level of agreement within BCS and MCS assessments collected from a population of active working EDDs during a routine veterinary visit. BCS and MCS were evaluated by four blinded graders: the EDD’s respective handler, an American College of Veterinary Sports Medicine and Rehabilitation (ACVSMR) resident, a diplomate of the ACVSMR (DACVSMR), and a primary care veterinarian of working dogs.

All fifty active federally owned EDDs that were scheduled for their routine veterinary visit were initially enlisted with approval from the canine unit supervisor and handler’s informed consent. Eligibility criteria required the EDDs to be healthy, adult (older than 1 year), and actively involved in explosive detection work. Forty-six handlers presented their EDDs for veterinary examination. Two EDDs were excluded for skipping a station and an additional EDD was excluded for completing the stations in the wrong order, resulting in a total of 43 EDDs included as study participants after thorough evaluation.

### Animal welfare and ethics

Veterinary Surgical Centers Rehabilitation (VSCR), a private veterinary facility, conducted a Department of Defense (DoD)-supported research study on March 21, 2023. The VSCR veterinary ethics committee reviewed and approved this study (protocol # 230321) on March 14th, 2023, determining it to be veterinary clinical research conducted on client-owned animals and exempt from Institutional Animal Care and Use Committee (IACUC). However, since this study required use of DoD facilities, equipment, and personnel, it also falls under the definition of research, development, test, and evaluation (RDT&E) supported by DoD. IACUC and Component oversight office approval are requirements of DoDI 3216.01 (33). Veterinary research utilizing client owned animals with informed consent is not under any legal requirement to comply with the Animal Welfare Act (34, 35). This study did not have any associated federal funding and therefore is not required to comply with the Public Health Policy (36). The American Veterinary Medical Association recommends that studies utilizing client-owned animals are reviewed by an IACUC or a Veterinary Clinical Studies Committee (VCSS) (37). Although the study did not receive pre-approval from an IACUC or the Component oversight office, informed consent was obtained from relevant officials and handlers and measures were taken to ensure the welfare of all animals involved. BCS and MCS are non-invasive hands-on assessments that do not cause any pain and are conducted routinely to assess fitness in semi-annual physical exams. As requested by the Army Animal Research Compliance and Oversight Office (ARCOO), a waiver to the IACUC and component oversight office approvals required by DoDI 3216.01 for publication of results was granted (Waiver Approval- Study #03212023) on April 14, 2024.

### Demographic characteristics

The EDD population, detailed in Table 1, consisted of 7 females (6 altered, 1 intact) and 36 males (4 altered, 32 intact). The median age was 5.3 years (ranging from 1.8 to 11.8 years), with a median weight of 32.2 kg (ranging from 20.2 to 47.9 kg) and a median withers height of 63.4 cm (ranging from 47.5 to 70.4 cm). This study encompassed various breeds, including 12 Belgian Malinois, 11 German Shepherds, 8 German Shorthaired Pointers, 5 Belgian Malinois Mixes, 3 Dutch Shepherds, 3 Labrador Retrievers, and 1 Labrador Retriever Mix, as outlined in Table 2.

**Table 1:** Sex, age, body weight, and withers height of EDD participants.

Demographic		Number
Sex	Male neutered	4
	Male intact	32
	Male total	36
	Female spayed	6
	Female intact	1
	Female total	7
Age (years)	Median	5.13
	Minimum	1.81
	Maximum	11.86
Body weight (kg)	Median	32.3
	Minimum	20.2
	Maximum	47.9
Withers height (cm)	Median	63.4
	Minimum	47.5
	Maximum	70.4

Demographic data, including sex and alteration status, as well as median, minimum, and maximum values for age, body weight, and withers height for the EDDs are recorded. These attributes provide a baseline for understanding the study population’s physical characteristics.

**Table 2:** Breed of EDD participants.

Breed	Number
Belgian malinois	12
Belgian malinois mix	5
Dutch shepherd	3
German shepherd	11
German shorthaired pointer	8
Labrador retriever	3
Labrador retriever mix	1
Total	43

Summary of the breeds of EDD participants, listing the number of dogs per breed. The data provides insight into breed-specific trends in the study population.

### Data collection

On March 21st, 2023, 43 EDD teams presented for their routine biannual veterinary visit and completed a rotation of four stations in the following order: Check-in and Handler Survey, Sports Medicine Dynamic Examination, Sports Medicine Complete Physical Examination, and Primary Care Examination. To maintain grader blinding, stations were physically separated (in different rooms).

All graders received laminated reference guides for BCS and MCS at the first of the four stations. At the check-in station (Station 1), each grader was provided with a formal introduction and didactic demonstration by a research and working animal veterinarian (JAB) who served as an instructor, not a grader, for the study. JAB led the graders through the reference guides for both BCS and MCS, which were provided to each handler to follow along as they were read aloud. JAB also helped to orient handlers to the key anatomical landmarks on their dogs for appropriate BCS assessment as well as demonstrated the face analogy using her own face to provide additional clarification for MCS grading.

For BCS assessment, graders received two nine-point BCS visual scales—one tailored for Labrador Retrievers by Nestlé Purina (7) and another for German Shepherds by Royal Canin (38) (see Supplementary Figures 1A,B). BCS scores of 4 or 5 were considered ‘ideal,’ with scores below 4 classified as ‘too thin’ and scores over 5 categorized as ‘too heavy.’

For MCS assessment, all graders were provided with a modified version of the WSAVA MCS scale (8), which included a visual aid illustrating a human face overlaid with numbers and descriptions relating to the scale (8) (see Supplementary Figure 2). MCS scores of 3 were labeled as ‘normal muscle condition,’ while scores of 2 indicated mild muscle atrophy, 1 signified ‘moderate muscle atrophy,’ and 0 indicated ‘significant muscle atrophy’ (8, 9).

The data collection protocol required each grader to use the provided reference guides while palpating and assigning Body Condition Score (BCS) and Muscle Condition Score (MCS) to the dogs, ensuring consistency across assessments. Handlers received one-on-one instructions with their dogs at each of the four stations. To ensure a collective understanding of the protocol, veterinary graders participated in a single instruction session before the study began. Each grader had access to the reference guides at their station throughout the process. After completing their assessments, graders recorded their responses on anonymized unique identifier (UID) cards, which were collected before the next dog was assessed. No additional tools, methods, or materials beyond the standard protocol were provided.

#### Station 1: Check-in and handler scoring

Station 1 was overseen by JAB, a research and working animal veterinarian. JAB checked in each EDD team, assigned a UID, confirmed handler consent to participate in the study, provided didactic instructions, and gathered an initial health history for each EDD. A licensed veterinary technician (LVT) distributed four UID cards to each handler, one for use at each station. Handlers then assessed their EDD’s body condition score through visual examination and palpation, followed by assigning a muscle condition score based on palpation. Afterward, the LVT weighed each EDD, recording the weight in kilograms. The UID card was collected, and the EDD teams advanced to Station 2.

#### Station 2: Resident sports medicine exam

Station 2 was led by an ACVSMR resident (KMC). Handlers presented their EDD’s UID card to KMC, who conducted a brief physical examination and followed the same protocol as other graders to assess and record the BCS and MCS. Additionally, the withers height was measured and recorded in centimeters (cm) using a standard yardstick (Hyper Tough™, Walmart Distribution Center, Bentonville, Arkansas), from a flat, level ground surface to the highest point of the shoulder blade on either side of the EDD. The corresponding UID card was collected, and the EDD teams proceeded to Station 3.

#### Station 3: Sports medicine complete physical examination

Station 3 was led by a DACVSMR (MWB). Handlers presented their EDD’s UID card to MWB, who performed a comprehensive examination and assessed and recorded BCS and MCS following the same protocol as the other graders. The corresponding UID card was collected, and the EDD teams proceeded to Station 4.

#### Station 4: Primary care examination

Station 4 was composed of a team of four primary care veterinarians from a practice that routinely provides care for the EDDs. The veterinarians were split into two examination lanes to provide patient care more efficiently. A list of all EDDs requiring care was provided to the veterinarians, and the order of EDD evaluation was based on post-time, with full veterinary care services provided after the BCS and MCS assessments. Although the four veterinarians worked collectively at Station 4, one veterinarian conducted the majority of the assessments, evaluating 26 of the 43 dogs, and was designated as the primary grader for this station. After the UID card was collected from the primary grader at this station, the veterinarians resumed their routine care for each respective EDD.

### Statistical analysis

The collected UID cards were processed, and the anonymized data was entered into a Microsoft Excel spreadsheet (Microsoft Corporation. (2018). Microsoft Excel). Excel data was uploaded into R Statistical Software for statistical analysis (v4.3.0; R Core Team 2023) (39). Both the BCS and MCS data were determined to not be normally distributed via Shapiro–Wilk test so Levene’s test for homogeneity of variance was used. Levene’s test showed that there was no difference in variance across grader, *F*(3) = 1.8, *p* = 0.149. This result met the assumptions for intraclass correlation analysis.

#### Analysis 1: Examining differences between graders

This analysis assessed whether any of the graders were systematically different from other graders in their determination of canine BCS.

A fully specified linear mixed-effects model was generated using the non-linear mixed effects (nlme) package (40) with BCS as the dependent variable, Grader as the fixed effect, and random intercepts of Dog, Breed, Age, Sex, and Alteration Status. However, this model did not converge, meaning that the statistical algorithm could not find a stable solution to fit the data. This often occurs when a model is too complex or includes too many variables. As a result, a simpler model with random intercepts for Dog, Breed, and Sex was used, which successfully converged and provided a better fit for the data.

This model was compared to reduced models with random intercepts for Dog and Breed only, and Dog only. Akaike Information Criterion (AIC) values were used to compare these models. The AIC is a measure that assesses how well a model fits the data while accounting for model complexity with lower values indicating a better fit. The model with Dog and Breed as random effects had the lowest AIC value, indicating that it was the best-fitting model for the data and was therefore selected for analysis.

The best-fitting linear mixed-effects model used BCS Score as the dependent variable, Grader as the fixed effect, and Dog and Breed as random intercepts to assess the effect of Grader identity on a given BCS score. The model was analyzed using a Type II Wald chi-square test to assess whether Grader as a variable has a significant impact on BCS Score. Post-hoc analyses with a Tukey adjustment comparing individual Graders to each other were done using the estimated marginal means (emmeans) package in R (41).

#### Analysis 2: Examining correlation between graders

This analysis assessed the extent to which the graders correlate with each other on their BCS ratings of dogs. Within-subject correlations were determined for all graders and pairs of graders using a linear mixed-effects model with BCS as the dependent variable, Grader as the fixed effect, and Dog as a random effect. Confidence intervals (95%) were estimated using a non-parametric bootstrap procedure. This analysis was done using the CorrMixed package in R (42).

## Results

### Descriptive statistics for BCS

The distribution of BCS ratings, as assigned by each grader, are illustrated in Table 3. The veterinary team and handlers assessed 67.44 and 74.4% of the EDD population, respectively, at an ideal BCS (4 or 5 out of 9); however, the graders scored different subsets of individual EDDs as ideal. Review of the BCS of all graders showed handlers significantly under-scored BCS compared to the three veterinary graders (*p* < 0.001).

**Table 3:** Distribution of EDDs by body condition score (BCS) and grader.

BCS (out of 9)	Handler	Primary care veterinarian	ACVSMR resident	DACVSMR	Veterinary team average (%)	BCS classification
1	0	0	0	0	0.00%	Too thin
2	3	0	0	0	0.00%	Too thin
3	4	2	2	0	3.10%	Too thin
4	18	11	10	12	25.58%	Ideal
5	14	22	12	20	41.86%	Ideal
6	3	7	13	6	20.16%	Too heavy
7	1	1	5	4	7.75%	Too heavy
8	0	0	1	1	1.55%	Too heavy
9	0	0	0	0	0.00%	Too heavy
Total	43	43	43	43	100%	

BCS ratings of the EDDs assigned by handlers and veterinary graders, as well as the percentage of dogs classified as too thin, ideal, or too heavy.

All four graders gave the same BCS score for 7 out of the 43 total EDDs (16.28%). For an additional 14 EDDs, three out of the four graders agreed on the BCS. In half of those cases, the grader that did not agree with the other three graders was the handler.

There are 15 dogs out of the total 43 (34.88%) for whom the grader disagreed by more than one BCS point. When the veterinary scores were examined alone, they only differed by more than one point on six dogs (13.95%).

### Model 1: Examining differences between graders

**Figure 1:**
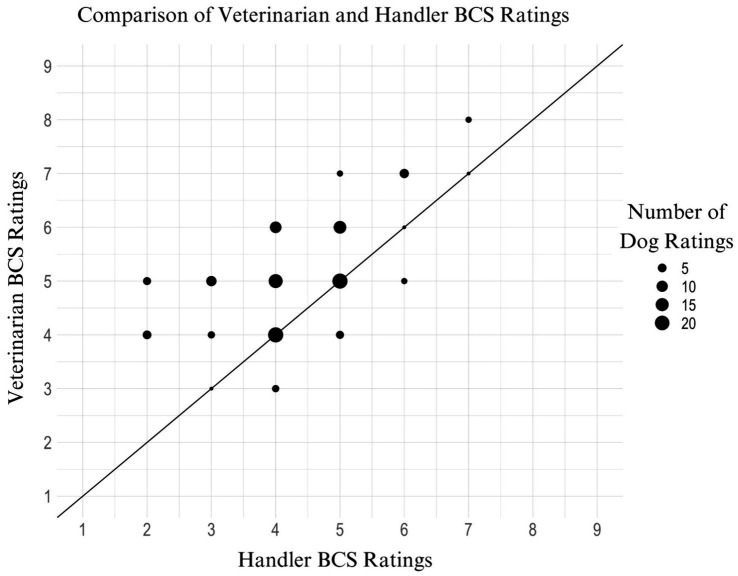
Scatter plot of veterinarian BCS rating vs. handler BCS rating for the same EDD. This scatter plot illustrates the relationship between BCS ratings assigned by handlers and veterinarians for the same EDDs. Each bubble represents the number of ratings at a specific BCS score, with bubble sizes ranging from 1 to 20 ratings, with reference example sizes of 5, 10, 15, and 20 shown in the legend. The line of best fit highlights a significant trend (*p* < 0.001) where handlers consistently scored BCS lower than veterinarians, indicating a systematic underscoring by handlers compared to veterinary assessments.

This model examined differences between graders in their evaluations of canine BCS. A significant main effect of Grader was observed (*X*^2^ = 46.92, *p* < 0.001), such that there were significant differences in graders’ scores. Subsequent post-hoc analyses, as detailed in Table 4, revealed that the scores assigned by handlers were significantly lower than those given by veterinarians (*p* < 0.001), while there were no significant differences among the scores assigned by different veterinarians. Figure 1 illustrates the BCS rating of the veterinarians compared to the corresponding handler BCS rating for the same EDD.

**Table 4:** Contrast values comparing scores for BCS.

Grader 1	Grader 2	Estimate	SE	df	*t*-ratio	*p*-value
Primary care veterinarian	DACVSMR	−0.256	0.149	127	−1.722	0.3165
Primary care veterinarian	ACVSMR resident	−0.349	0.149	127	−2.348	0.0927
Primary care veterinarian	Handler	0.558	0.149	127	3.758	0.0015*
DACVSMR	ACVSMR resident	−0.093	0.149	127	−0.626	0.9235
DACVSMR	Handler	0.814	0.149	127	5.48	<0.001*
ACVSMR resident	Handler	0.907	0.149	127	6.106	<0.001*

The estimate, standard error (SE), degrees of freedom (df), *t*-ratio, and *p*-value are recorded for each pair of graders. The estimate value refers to the difference of marginal means between grader 1 and grader 2, which is the average difference in their BCS scores. The *t*-ratio refers to the difference between sample means divided by the standard error of the difference; if the absolute value of the *t*-ratio scores is high, it will generally result in a higher *p*-value. This analysis highlights the statistical differences in BCS scoring between graders, with a particular focus on the significant differences between handlers and veterinarians. *p < 0.05.

### Model 2: Examining correlation between graders

This set of models examined the correlation between all graders and then between each pair of graders in their assessments of canine BCS which can be found in Table 5. The estimated correlation coefficient for all graders was found to be 0.62, with a 95% confidence interval ranging from 0.45 to 0.72. When the handlers’ scores were excluded, the estimated correlation coefficient for all veterinarians increased to 0.66, with a 95% confidence interval ranging from 0.49 to 0.77.

**Table 5:** Correlation coefficients (*R*^2^) for pairs of graders for BCS.

Grader 1	Grader 2	*R*^2^	95% Confidence interval
Primary care veterinarian	DACVSMR	0.52	[0.18, 0.69]
Primary care veterinarian	ACVSMR resident	0.53	[0.28, 0.73]
Primary care veterinarian	Handler	0.34	[0.05, 0.55]
DACVSMR	ACVSMR resident	0.88	[0.79, 0.93]
DACVSMR	Handler	0.68	[0.46, 0.82]
ACVSMR resident	Handler	0.65	[0.45, 0.78]

The level of agreement between pairs of graders is demonstrated by the correlation coefficients (represented by *R*^2^). The higher the correlation coefficient on a scale of 0 to 1, the stronger the association. This table illustrates the degree of consistency in BCS evaluations among different graders.

Variance partition coefficients were calculated to assess the proportion of the variance explained by the random effect variables used in the model; these coefficients result in Intraclass Correlation Coefficient (ICC) values, which measure the degree of agreement or consistency between measurements and were used in our study to assess interrater reliability (43). The ICC for Dog was 0.62 and the ICC for Breed was 0.45, meaning that individual Dog explains 62% of the variance in the data, and Breed explains 45% of the variance. Additionally, the combination of these two random effects explains 69% of the variability in the data, further justifying the use of a multilevel model given the high level of variance explained by these variables.

### Descriptive statistics for MCS

The MCS given to the EDD population by each grader is presented in Table 6. The handlers graded 86.05% (*n* = 37) of EDDs as having normal MCS (3 out of 3) versus 70.54% by the veterinary team. Mild muscle atrophy (MCS 2 out of 3) was assessed in 13.95% (*n* = 6) of EDDs by the handlers versus an average of 29.46% by the veterinary team. No EDDs were evaluated to have moderate muscle atrophy (MCS 1 out of 3) or marked muscle atrophy (MCS 0 out of 3) by any grader.

**Table 6:** Distribution of EDDs by muscle condition score (MCS) and grader.

MCS (out of 3)	Handler *n* (%)	Primary care veterinarian *n* (%)	ACVSMR resident *n* (%)	DACVSMR *n* (%)	Veterinary team average (%)	MCS classification
0	0 (0.00%)	0 (0.00%)	0 (0.00%)	0 (0.00%)	0.00%	Marked muscle atrophy
1	0 (0.00%)	0 (0.00%)	0 (0.00%)	0 (0.00%)	0.00%	Moderate muscle atrophy
2	6 (13.95%)	5 (11.63%)	11 (25.48%)	22 (48.84%)	29.46%	Mild muscle atrophy
3	37 (86.05%)	38 (88.37%)	32 (74.42%)	21 (51.16%)	70.54%	Normal musculature
Total	43	43	43	43	100%	

The distribution of MCS ratings assigned by both handlers and veterinary graders, as well as the percentages for each MCS category (normal muscle condition, mild muscle atrophy, etc.) are recorded. The table highlights the differences between handler and veterinary assessments, illustrating any discrepancies in muscle condition scoring. These comparisons provide insight into the level of consistency between handlers’ and veterinarians’ evaluations of muscle condition in working dogs.

## Discussion

To the authors’ knowledge, this is the first study to assess working canine handlers’ evaluations of their dogs’ BCS and MCS and to compare them to veterinarians. The results of our study demonstrated there was no significant difference amongst veterinary professionals in BCS scoring, regardless of their level of expertise. However, the results also revealed a significant disparity between handlers and veterinary graders in which handlers scored their canine partners’ BCS significantly lower than veterinary graders (Table 4). This underscoring of BCS is consistent with previous research on owner assessment of their own dogs’ BCS (27–32). Despite handlers’ specialized training in working with their canine partners, variations in expertise or training related to BCS assessment may contribute to the underestimation of BCS scoring by handlers compared to veterinary graders. Previous research by Gille et al. (44) highlighted the challenges individuals face in assigning accurate BCS if unfamiliar with BCS scales. While information regarding handlers’ experiences and familiarity with BCS scales was unavailable during data collection, it is possible handlers lacked prior exposure and/or experience to the scales used in this study, affecting their ability to assign BCS scores accurately or in agreement with veterinary graders.

This study also evaluated the level of agreement between graders in the assessment of BCS as illustrated in Table 5. The agreement in BCS assessment between all four graders was 0.62, which indicated a moderate agreement. However, the highest level of agreement, at 0.88 (Table 5), occurred between the ACVSMR resident and the DACVSMR, indicating good agreement. This suggests a stronger consensus between these two graders than among all four graders collectively. The higher agreement noted between the ACVSMR resident and the DACVSMR could be explained by similar training in BCS assessment, along with the additional emphasis on BCS within the specialty of canine sports medicine.

Despite the moderate to good level of agreement on BCS among the graders in our study, the handlers consistently underscored the BCS of their respective canine partners compared to the three veterinary graders. This discrepancy may suggest that veterinarians have an increased familiarity with BCS assessment charts and experience evaluating fit, healthy dog populations, causing them to score BCS more similarly. The three veterinary professionals evaluated the EDDs without familiarity bias, as they had no prior attachment to the dogs during data collection. This lack of familiarity bias likely contributed to the observed agreement among the veterinary graders in BCS evaluation. Furthermore, veterinarians specializing in canine sports medicine may have increased utilization of BCS assessment practices in working dogs, potentially resulting in better agreement among this group. While proficiency in BCS scales is crucial for identifying potential health issues in working dogs, including EDDs, further research is needed to assess the impact of additional training on inter-rater agreement.

Our study enrolled only healthy, active-duty EDDs, with three out of four graders assessing 32–38 dogs (74.42–88.37%) as having normal muscle condition. In our study, the DACVSMR assessed 48.84% (*n* = 22) of the EDDs to have mild muscle atrophy. This could be due to the DACVSMR’s extensive experience in utilizing MCS in the assessment of working dogs. The current unipolar MCS scale is designed for disease detection (8, 10, 20, 24, 45) and therefore limited in identifying positive muscle development. A bipolar MCS scale which encompasses not only the absence of muscle atrophy, but also incorporates varying degrees of muscle development, could be considered. Ramos et al. (4) proposed such a scale, grading MCS out of a total of 5. In their framework, an MCS of 4 indicates toned musculature, ideal for athletic dogs (sporting dogs and most working dogs), and an MCS of 5 signifies hypertrophic muscle or ‘double muscling,’ which could be suitable for certain specialized working dogs or represent a pathologic change. A bipolar MCS scale would allow for a more complete assessment of a canine’s muscular health and bring it into further alignment with the BCS framework.

While our study design prevented leakage of graders’ assessments to the other graders during the study, handlers’ prior experiences were not investigated in this study, but may have influenced handler responses. Handlers are frequently exposed to public, trainer, and veterinary comments on the body condition of their dogs. Previous comments or assessments, particularly if associated with negative societal connotations, may have contributed to a conformity bias and influenced how the handlers evaluated their EDDs in our study.

There are limitations to our study. One limitation is the potential bias introduced during data collection. As stated in the Materials and Methods, all graders were given laminated reference guides of BCS and MCS (see Supplementary Figures 1A,B) which included descriptive language and colors. The BCS scales used include words such as ‘obese,’ ‘overweight,’ ‘too heavy,’ and ‘too thin’ with red and yellow coloration. Such words can carry more negative connotations, as demonstrated in a 2013 study by Puhl et al. (46). Less experienced graders may have been influenced by these terms, potentially skewing their assessments. Furthermore, the use of specific colors, such as green for an ‘ideal’ BCS or the addition of a lighter background color to highlight the ‘ideal’ scores on a BCS chart, may have encouraged graders to select certain scores for an EDD, regardless of their initial evaluation of the dog’s body condition. Conversely, red and yellow or darker background colors for ‘obese,’ ‘overweight,’ and ‘too heavy’ may have encouraged graders to avoid selecting certain scores. The use of less common terms, like ‘atrophy’ instead of ‘loss’ on the MCS chart may have biased graders less familiar with these terms, leading to unintentional misinterpretation rather than evaluating the amount of lean muscle tissue accurately. To mitigate potential bias, future charts could be printed in black and white, distributed without descriptive wording, or be modified to include more common wording allowing graders to assess the dogs solely based on visual evaluation and palpation as intended.

It is also recognized that handlers are biased toward their own dog’s performance and are more critical of other dogs (47). In our study, handlers graded the highest percentage of dogs as ideal BCS compared to the veterinary team (74.4 and 67.44%, respectively). This positive bias toward a handler’s own dog may have contributed to our result and future studies could test this by having a handler or handlers assess a group of working dogs that excluded their canine partner to determine if the results would differ from those in this study.

While our study focused on assessing the level of agreement for BCS and MCS within a healthy, active-duty EDD population, further investigations into diverse working dog populations are necessary to assess the relevance of our findings. Examining other working dog cohorts could uncover additional variations in inter-rater agreement, especially considering the potential heterogeneity in these populations. These variations may stem from differences in breeds, tasks, environmental conditions, and overall health status among the different populations of working dogs. Furthermore, expanding the study to include additional graders, such as trainers familiar with the specific tasks and physical requirements of working dogs, or employing different handler/canine pairings, could help us to understand the influence of expertise or specialized training in BCS and MCS assessment. By incorporating perspectives from various personnel involved in the care and training of working dogs, we can gain a more comprehensive understanding of the factors influencing inter-rater agreement and improve the accuracy and reliability of BCS and MCS evaluations across diverse working dog populations.

## Data Availability

The original contributions presented in the study are included in the article/Supplementary material, further inquiries can be directed to the corresponding author.

## References

[1] MacLean EL Hare B. Enhanced selection of assistance and explosive detection dogs using cognitive measures. Front Vet Sci. (2018) 5:408876. doi: 10.3389/fvets.2018.00236 PMC6180148 30338264

[2] Lazarowski L Waggoner LP Krichbaum S Singletary M Haney P Rogers B. Selecting dogs for explosives detection: behavioral characteristics. Front Vet Sci. (2020) 7:597. doi: 10.3389/fvets.2020.00597 33088829 PMC7493654

[3] Otto CM Darling T Murphy L Ng Z Pierce B Singletary M. 2021 AAHA working, assistance, and therapy dog guidelines. J Am Anim Hosp Assoc. (2021) 57:253–77. doi: 10.5326/JAAHA-MS-7250, PMID: 34710214

[4] Ramos MT Farr BD Otto CM. Sports medicine and rehabilitation in working dogs. Vet Clin North Am Small Anim Practice. (2021) 51:859–76. doi: 10.1016/j.cvsm.2021.04.005, PMID: 34059260

[5] Salt C Morris PJ German AJ Wilson D Lund EM Cole TJ. Growth standard charts for monitoring bodyweight in dogs of different sizes. PLoS One. (2017) 12:e0182064. doi: 10.1371/journal.pone.0182064, PMID: 28873413 PMC5584974

[6] German AJ Morgan LE. How often do veterinarians assess the bodyweight and body condition of dogs? Vet Rec. (2008) 163:503–5. doi: 10.1136/vr.163.17.503, PMID: 18953073

[7] Laflamme DP. Development and validation of a body condition score system for dogs. Canine Practice. (1997) 22:10–5.

[8] WSAVA Nutritional Assessment Guidelines Task Force Members Freeman L Becvarova I Cave N MacKay C Nguyen P. WSAVA Nutritional Assessment Guidelines. J Small Anim Pract. (2011) 52:385–96. doi: 10.1111/j.1748-5827.2011.01079.x, PMID: 21649660

[9] Michel KE Anderson W Cupp C Laflamme DP. Correlation of a feline muscle mass score with body composition determined by dual-energy X-ray absorptiometry. Br J Nutr. (2011) 106:S57–9. doi: 10.1017/S000711451100050X, PMID: 22005437

[10] Freeman LM Sutherland-Smith J Prantil LR Sato AF Rush JE Barton BA. Quantitative assessment of muscle in dogs using a vertebral epaxial muscle score. Can J Vet Res (2017): 1;81:255–260, PMID: 29081582 PMC5644455

[11] Shepherd M. Canine and feline obesity management. Vet Clin. (2021) 51:653–67. doi: 10.1016/j.cvsm.2021.01.005, PMID: 33653534

[12] Kealy RD Lawler DF Ballam JM Mantz SL Biery DN Greeley EH. Effects of diet restriction on life span and age-related changes in dogs. J Am Vet Med Assoc. (2002) 220:1315–20. doi: 10.2460/javma.2002.220.1315, PMID: 11991408

[13] German AJ. The growing problem of obesity in dogs and cats. J Nutr. (2006) 136:1940S–6S. doi: 10.1093/jn/136.7.1940S, PMID: 16772464

[14] Lund EM Armstrong PJ Kirk CA Klausner JS. Prevalence and risk factors for obesity in adult dogs from private US veterinary practices. Int J Appl Res Vet Med. (2006) 4:177.

[15] German AJ Ryan VH German A Wood IS Trayhurn P. Obesity, its associated disorders and the role of inflammatory adipokines in companion animals. Vet J. (2010) 185:4–9. doi: 10.1016/j.tvjl.2010.04.004, PMID: 20472476

[16] Gazit I Terkel J. Domination of olfaction over vision in explosives detection by dogs. Appl Anim Behav Sci. (2003) 82:65–73. doi: 10.1016/S0168-1591(03)00051-0

[17] Manens J Ricci R Damoiseaux C Gault S Contiero B Diez M. Effect of body weight loss on cardiopulmonary function assessed by 6-minute walk test and arterial blood gas analysis in obese dogs. J Vet Intern Med. (2014) 28:371–8. doi: 10.1111/jvim.12260, PMID: 24351032 PMC4858022

[18] Otto CM Hare E Nord JL Palermo SM Kelsey KM Darling TA. Evaluation of three hydration strategies in detection dogs working in a hot environment. Front Vet Sci. (2017) 4:174. doi: 10.3389/fvets.2017.00174 29124059 PMC5662554

[19] Aviles-Rosa E Schultz J Maughan MN Gadberry JD DiPasquale DM Farr B. A canine model to evaluate the effect of exercise intensity and duration on olfactory detection limits: the running nose. Front Allergy. (2024) 5:1367669. doi: 10.3389/falgy.2024.1367669, PMID: 38784159 PMC11111909

[20] Freeman LM Michel KE Zanghi BM Vester Boler BM Fages J. Evaluation of the use of muscle condition score and ultrasonographic measurements for assessment of muscle mass in dogs. Am J Vet Res. (2019) 80:595–600. doi: 10.2460/ajvr.80.6.595 31140851

[21] Santarossa A Parr JM Verbrugghe A. The importance of assessing body composition of dogs and cats and methods available for use in clinical practice. J Am Vet Med Assoc. (2017) 251:521–9. doi: 10.2460/javma.251.5.521, PMID: 28828948

[22] Burkholder WF. Use of body condition scores in clinical assessment of the provision of optimal nutrition. J Am Vet Med Assoc. (2000) 217:650–4. doi: 10.2460/javma.2000.217.650, PMID: 10976293

[23] German AJ Holden SL Moxham GL Holmes KL Hackett RM Rawlings JM. A simple, reliable tool for owners to assess the body condition of their dog or cat. J Nutr. (2006) 136:2031S–3S. doi: 10.1093/jn/136.7.2031S 16772488

[24] Baldwin KE Bartges JW Buffington T Freeman LM Grabow M Legred J. AAHA nutritional assessment guidelines for dogs and cats. J Am Anim Hosp Assoc. (2010) 46:285–96. doi: 10.5326/0460285, PMID: 20610704

[25] Mawby DI Bartges JW D’Avignon A Laflamme DP Moyers TD Cottrell T. Comparison of various methods for estimating body fat in dogs. J Am Anim Hosp Assoc. (2004) 40:109–14. doi: 10.5326/0400109, PMID: 15007045

[26] Chun JL Bang HT Ji SY Jeong JY Kim M Kim B. A simple method to evaluate body condition score to maintain the optimal body weight in dogs. J Anim Sci Technol. (2019) 61:366–70. doi: 10.5187/jast.2019.61.6.366, PMID: 31844547 PMC6906133

[27] Eastland-Jones RC German AJ Holden SL Biourge V Pickavance LC. Owner misperception of canine body condition persists despite use of a body condition score chart. J Nutr Sci. (2014) 3:e45. doi: 10.1017/jns.2014.25, PMID: 26101613 PMC4473163

[28] Teixeira FG Queiroz MR Oba PM Olivindo RFG Ernandes MC Duarte CN. Brazilian owners perception of the body condition score of dogs and cats. BMC Vet Res. (2020) 16:463. doi: 10.1186/s12917-020-02679-8, PMID: 33246455 PMC7694915

[29] Courcier EA Mellor DJ Thomson RM Yam PS. A cross sectional study of the prevalence and risk factors for owner misperception of canine body shape in first opinion practice in Glasgow. Prev Vet Med. (2011) 102:66–74. doi: 10.1016/j.prevetmed.2011.06.010, PMID: 21820746

[30] Kluess HA Jones R Lee-Fowler TM. Perceptions of body condition, diet and exercise by sports dog owners and pet dog owners. Animals. (2021) 11:1752. doi: 10.3390/ani11061752, PMID: 34208293 PMC8230856

[31] Yam PS Naughton G Butowski CF Root A. Inaccurate assessment of canine body condition score, bodyweight, and pet food labels: a potential cause of inaccurate feeding. Vet Sci. (2017) 4:30. doi: 10.3390/vetsci4020030, PMID: 29056689 PMC5606605

[32] White GJ Hobson-West P Cobb KM Craigon J Hammond R Millar K. Canine obesity: is there a difference between veterinarian and owner perception? J Small Anim Pract. (2011) 52:622–6. doi: 10.1111/j.1748-5827.2011.01138.x, PMID: 22017760

[33] Department of Defense (DoD). DoD Instruction (DoDI) 3216.01 Use of animals in dod conducted and supported research and training [Internet]. Office of the Under Secretary of Defense for Research and Engineering. (2019).

[34] United States Code. Title 7 (Agriculture), Chapter 54 (Transportation, Sale, and Handling of Certain Animals), Sections 2131–2159. Office of the Law Revision Counsel, U.S. House of Representatives. (1985). Available at: https://uscode.house.gov

[35] Code of Federal Regulations. Title 9 (Animals and Animal Products), Chapter 1 (Animal and Plant Health Inspection Service, Department of Agriculture), Subchapter A (Animal Welfare), Parts 1-4. U.S Government Publishing Office. (2023).

[36] National Institutes of Health, Office of Laboratory Animal Welfare. Public Health Service Policy on Humane Care and Use of Laboratory Animals. U.S. Department of Health and Human Services. (2015). Available at: https://olaw.nih.gov

[37] American Veterinary Medical Association Policy. Establishment and use of veterinary clinical studies committees. American Veterinary Medical Association, Schaumburg, IL. Available at: https://www.avma.org/resources-tools/avma-policies/establishment-and-use-veterinary-clinical-studies-committees (Accessed November 3, 2023).

[38] Body Condition Score Charts Cat and Small Dog. (2013). Available at: https://www.royalcanin.co.uk/wp-content/uploads/2017/02/BCS-chart-03.12.13.pdf (Accessed April 16, 2023).

[39] R Core Team. R: A language and environment for statistical Computing. Vienna, Austria: R Foundation for Statistical Computing (2023).

[40] Pinheiro J. Bates D. R Core Team. (2023). *nlme: Linear and nonlinear mixed effects models* (R package version 3.1–163). Available at: https://CRAN.R-project.org/package=nlme

[41] Lenth R. (2024). *emmeans: Estimated marginal means, aka least-squares means* (R package version 1.10.2). Available at: https://CRAN.R-project.org/package=emmeans

[42] Van der Elst W. Molenberghs G. Hilgers D. Heussen N. (2022). *CorrMixed: Estimate correlations between repeatedly measured endpoints (e.g., reliability) based on linear mixed-effects models* (R package version 1.1). Available at: https://CRAN.R-project.org/package=CorrMixed 10.1002/pst.1787 27681820

[43] Koo TK Li MY. A guideline of selecting and reporting intraclass correlation coefficients for reliability research. J Chiropr Med. (2016) 15:155–63. doi: 10.1016/j.jcm.2016.02.012 27330520 PMC4913118

[44] Gille S Fischer H Lindåse S Palmqvist L Lärka J Wolf S. Dog owners’ perceptions of dog body composition and effect of standardized education for dog owners on body condition assessment of their own dogs. Vet Sci. (2023) 10:447. doi: 10.3390/vetsci10070447, PMID: 37505852 PMC10386090

[45] Freeman LM. Cachexia and sarcopenia: emerging syndromes of importance in dogs and cats. J Vet Intern Med. (2012) 26:3–17. doi: 10.1111/j.1939-1676.2011.00838.x, PMID: 22111652

[46] Puhl R Peterson JL Luedicke J. Motivating or stigmatizing? Public perceptions of weight-related language used by health providers. Int J Obes. (2013) 37:612–9. doi: 10.1038/ijo.2012.110 22777543

[47] Clark CC Sibbald NJ Rooney NJ. Search dog handlers show positive bias when scoring their own dog’s performance. Front Vet Sci. (2020) 7:612. doi: 10.3389/fvets.2020.00612, PMID: 33195498 PMC7533607

