# Supplementary figures and images for: Interobserver variability of assessing body condition scores and muscle condition scores in a population of 43 active working explosive detection dogs
Source: Front Vet Sci. 2024 Oct 17;11:1431855. doi: 10.3389/fvets.2024.1431855 (PMC11525006; doi:10.3389/fvets.2024.1431855)

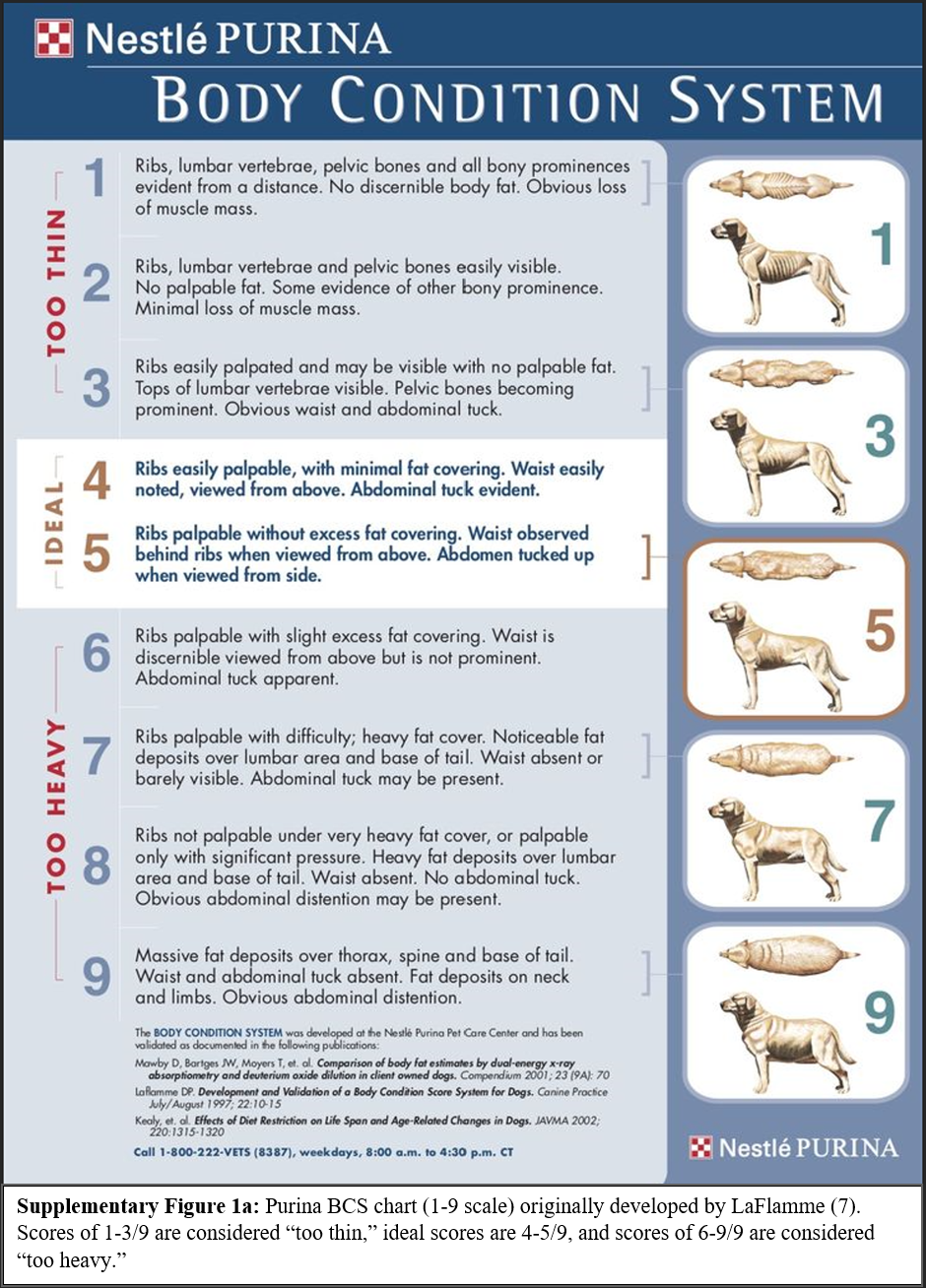

Supplement: Supplementary file 1 [file Image_1.png]

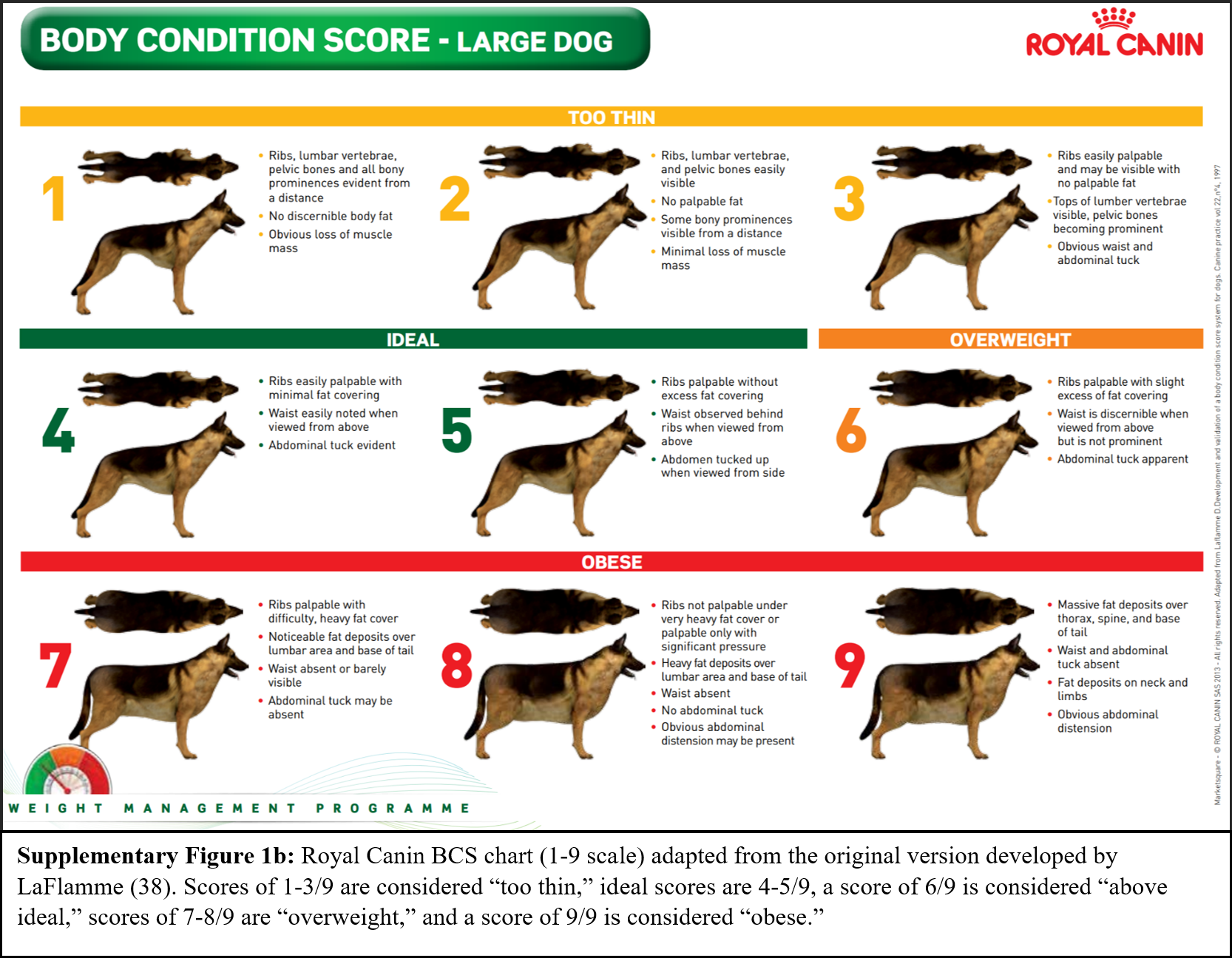

Supplement: Supplementary file 2 [file Image_2.png]

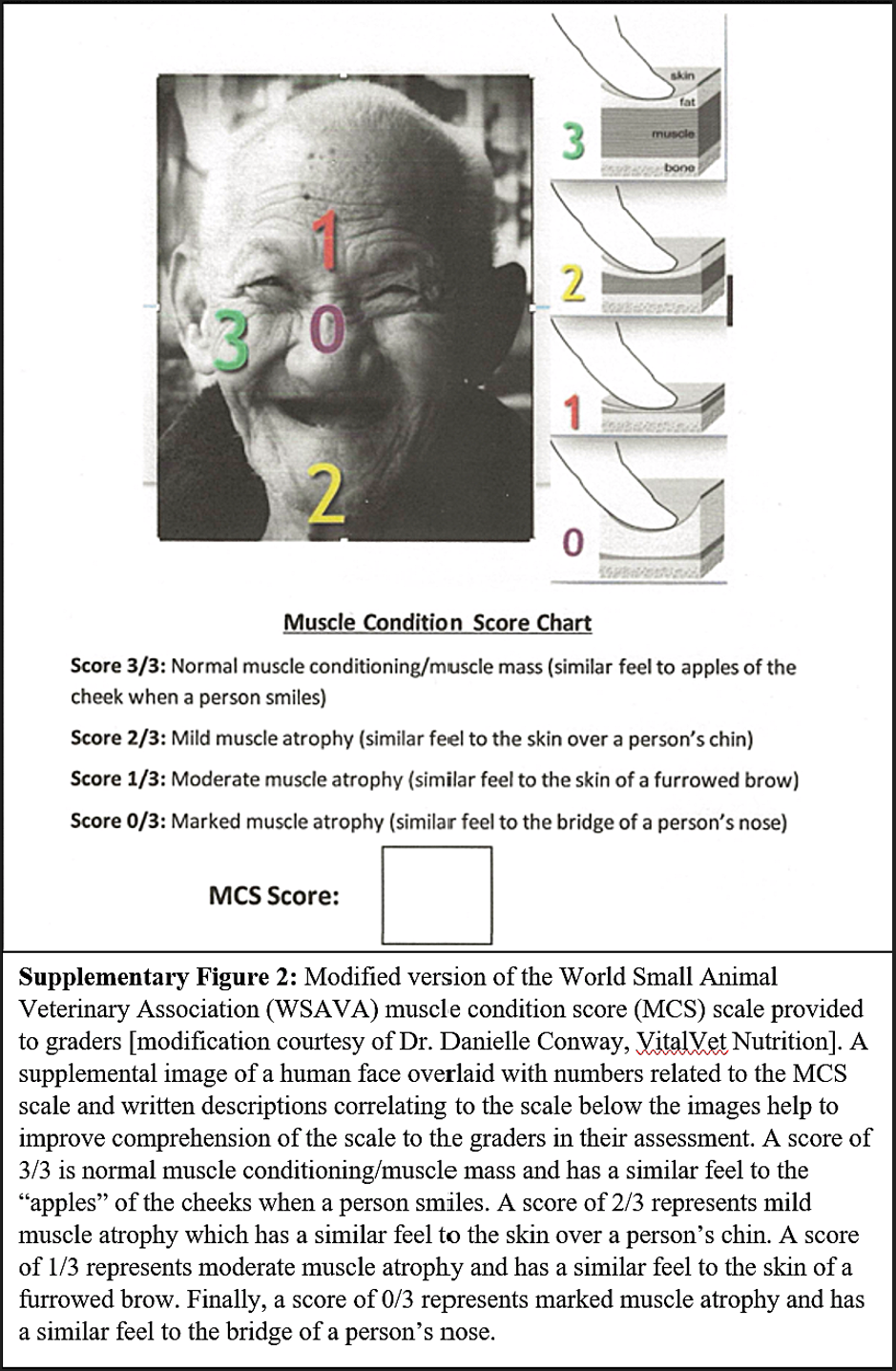

Supplement: Supplementary file 3 [file Image_3.png]
